# Management of an Entrapped Epidural Catheter

**DOI:** 10.7759/cureus.56919

**Published:** 2024-03-25

**Authors:** Justin Hruska, Melanie Darke

**Affiliations:** 1 Anesthesiology, Detroit Medical Center, Detroit, USA; 2 Anesthesiology, Ascension St. John Hospital, Detroit, USA

**Keywords:** epidural anesthesia, postoperative pain management, neuraxial analgesia, device entrapment, epidural catheter complication

## Abstract

Epidural catheters are seldom challenging to remove from patients. The occurrence of knotting in an epidural catheter, resulting in entrapment, is an uncommon complication of epidural catheterization. There is the risk of significant morbidity with a retained catheter, with the potential for infection or nerve injury. This report describes the techniques used in a case where surgical removal of an entrapped epidural was required and discusses other potential strategies for the successful management of this type of complication. In this case, a low thoracic epidural catheter that was inserted into a 68-year-old male for post-operative analgesia proved challenging to remove. After multiple attempts to remove the catheter, a lumbar CT scan and neurosurgical evaluation were obtained. The neurosurgical team decided to perform a right thoracic hemilaminectomy to remove the entrapped catheter. This surgery revealed a knot near the distal tip of the catheter, which likely caused the entrapment of the catheter in the epidural space.

## Introduction

Epidural anesthesia is a highly effective and well-tolerated technique for pain management after abdominal surgery [1]. Complications related to epidural catheter insertion are rare and may include catheter breakage, entrapment, and knotting [1]. Various techniques have been described for safe removal of the catheter, including change of patient position, slow traction, continued traction over time, and injection of saline [2]. If conservative measures fail in the removal of the catheter, it is recommended to consider radiological imaging and neurosurgical evaluation [2].

## Case presentation

A 68-year-old male weighing 90 kg (BMI 30.8 kg/m^2^) presented to the operating room for exploratory laparotomy with gastrectomy and Roux-en-Y gastrojejunostomy. The patient had a history of obesity, hypertension, insulin-dependent diabetes mellitus, and hyperlipidemia.

Prior to surgery, an anesthesiologist inserted an epidural catheter using a midline approach at the T11-12 intervertebral space with the patient in a sitting position. A 17-gauge Tuohy needle was used with the loss of resistance with saline technique. On the second attempt, the epidural space was identified at a depth of 7 cm, and the catheter was easily advanced through the epidural needle and fixed at 15 cm on the skin. A test dose of 3 mL of lidocaine 1.5% with epinephrine (1:200,000) indicated no evidence of intravascular or subarachnoid catheter placement. Both anesthesia and surgery were uneventful, and the epidural catheter was infused with 0.0625% bupivacaine with hydromorphone 5 μg/mL to provide effective postoperative pain relief.

The nursing staff attempted to remove the epidural catheter 72 hours after the surgery, following the institutional practice. The catheter was removed easily until the 5-cm mark became visible at the skin, where significant resistance was encountered. An anesthesiology resident tried to remove the catheter multiple times. The resident attempted to remove the epidural catheter with the patient in various positions, including lateral position with lumbar flexion, extension, and lateral flexion to each side, but all attempts were unsuccessful. To confirm the catheter patency, 5 mL of 0.9% normal saline bolus was injected through the catheter, but it failed to aid the removal process. Given the circumstances, the decision was made to leave the catheter in place and obtain a lumbar CT scan and neurosurgical evaluation.

**Figure 1:**
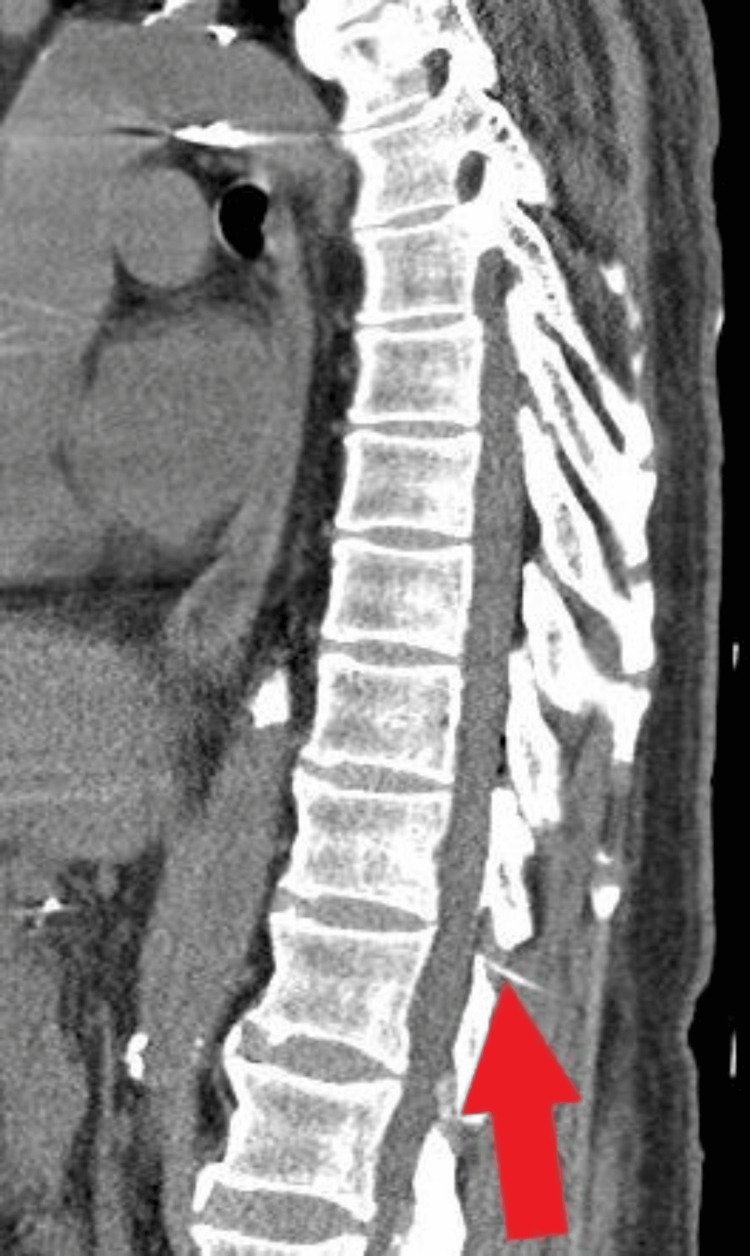
Sagittal CT scan of the thoracic and lumbar spine, with red arrow pointing to the epidural catheter located between the lamina of T11 and T12.

**Figure 2:**
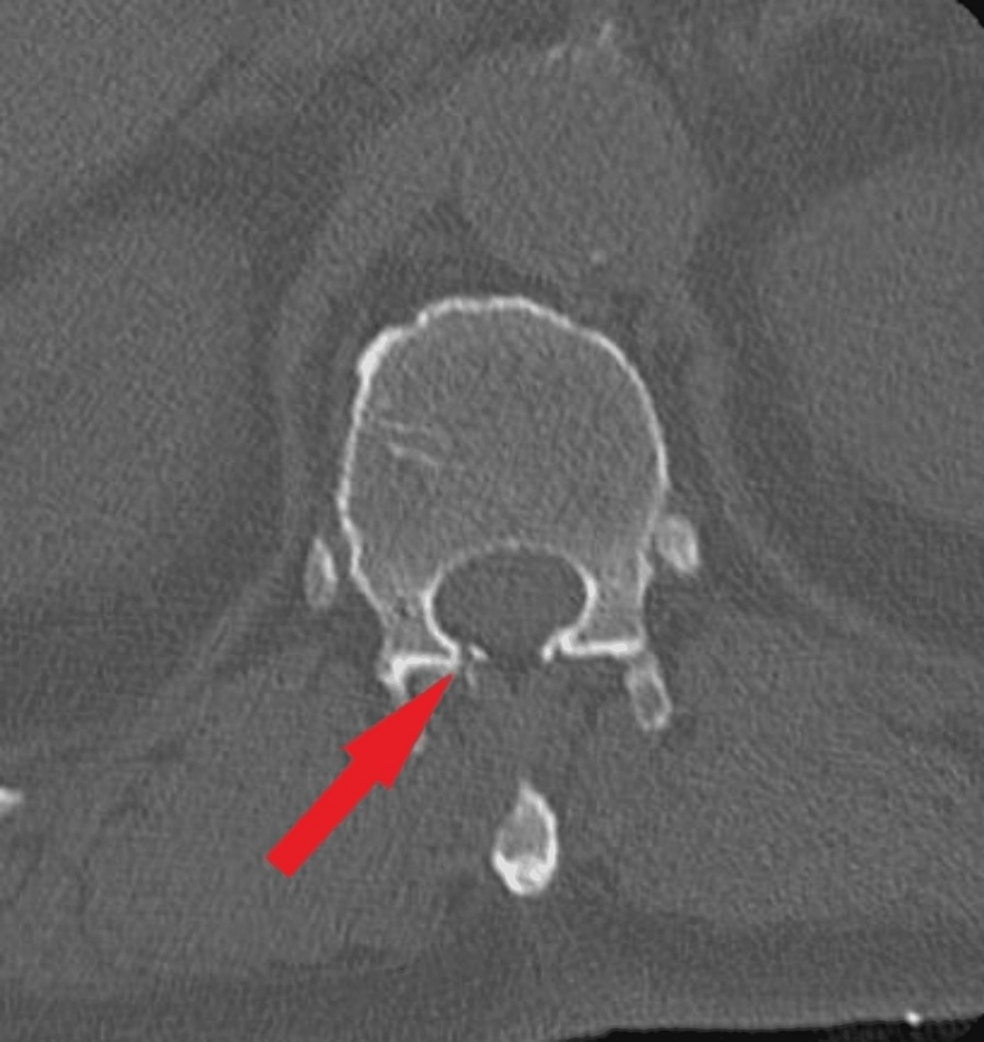
Axial CT scan of lumbar spine, with the red arrow pointing to the epidural catheter with the tip in the epidural space at T11/T12.

The CT scan showed the epidural catheter enters on the right between the lamina of T11 and T12, with its distal tip positioned in the epidural space at T11/T12 (Figures 1, 2).

**Figure 3:**
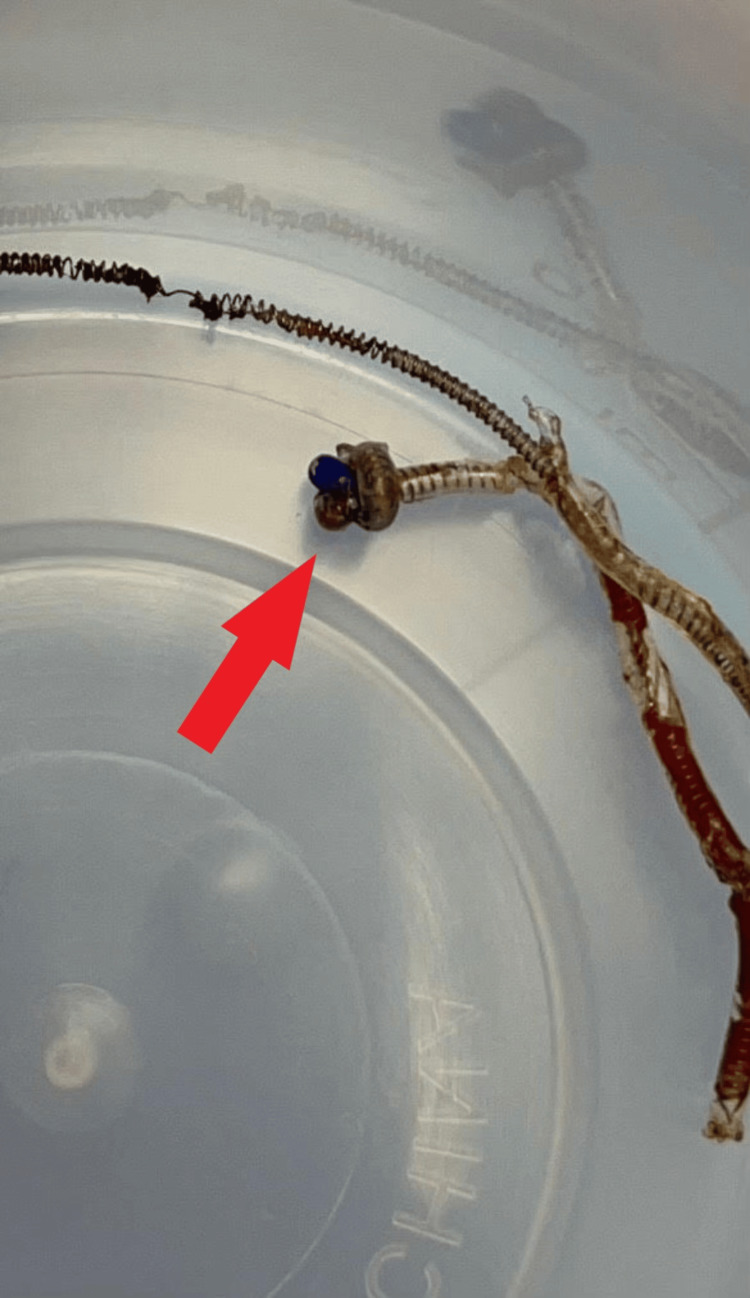
The surgically removed epidural catheter, with the red arrow pointing to the knot at the tip of the catheter.

The decision was made to surgically remove the catheter via a right thoracic hemilaminectomy under general anesthesia, which was performed 72 hours after the entrapped catheter was identified. During the surgery, the distal tip of the catheter was noted to have a knot that was the likely cause of its entrapment in the epidural space (Figure 3).

The patient was discharged home three days after the right thoracic hemilaminectomy surgery, and there was no additional follow-up data.

## Discussion

Recognized complications associated with epidural catheter placement encompass unsuccessful placement, inadvertent intrathecal or intravascular placement, catheter damage, knot formation, and challenging removal [1]. The incidence of difficulty in epidural catheter withdrawal is around one in 20,000 to 30,000 [3]. Formation of loops or knots during catheter insertion is a common reason for entrapped catheters [2], and the risk is increased with excessive catheter threading into the epidural space [4]. The ideal length of insertion, nonetheless, remains a matter of debate. Recommendations suggest that the catheter insertion length should not surpass 5 cm, with an optimal range of less than 3-4 cm to mitigate the risk of knot formation [5,6].

During insertion, an epidural catheter may encounter anatomical obstructions, leading to deflection or curling back on itself. Twisting of the catheter may occur along nerve roots, blood vessels, lumbar fascia, posterior vertebral arches, vertebral processes, or facet joints [4]. Furthermore, it has been proposed that epidural catheters utilized for extended durations (>24 hours) may develop resistance to traction due to inflammation, subsequent fibrosis, or catheter migration [7].

Complications arising from catheter entrapment include breaking of the catheter, hematoma formation, epidural site infection, and the potential for neurological deficits [5].

Recommended techniques in cases of resistance to withdrawal of an epidural catheter include gentle constant traction in the position of epidural insertion or constant traction in varying postures such as lateral decubitus [8]. It has been reported that a seated position may exert increased resistance to the removal of an epidural catheter by as much as 2.5-fold [8]. Our patient was obese, had recent abdominal surgery, and encountered difficulty assuming an adequate position for the removal of the catheter.

If traction is unsuccessful in removing the epidural, normal saline injection through the epidural is suggested as a next step. This may also aid in diagnosing a true knot in the catheter [2].

In cases of excessive catheter stretch (typically after multiple attempts at traction) or increased pain associated with the attempts, traction should be stopped at once and imaging of the catheter should be considered [2].

**Figure 4:**
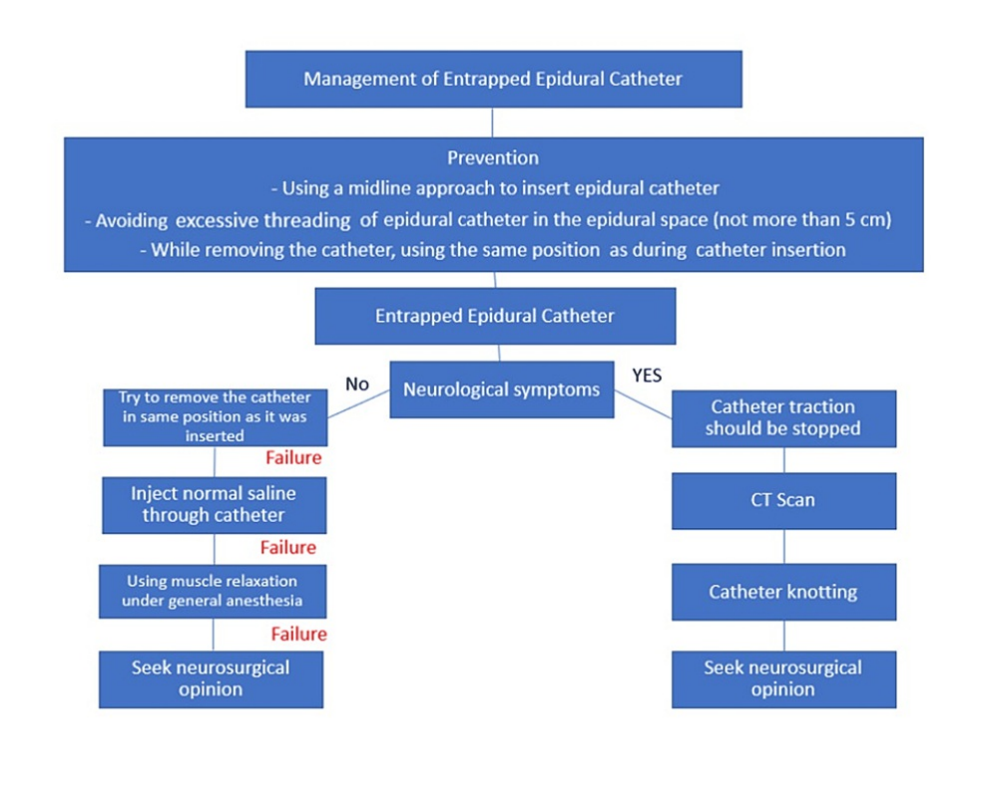
Algorithm of the management of an entrapped epidural catheter Image Credits: Justin Hruska

Epidural catheters resistant to conventional extraction methods may necessitate removal under either local or general anesthesia. While local anesthesia mitigates patient discomfort, general anesthesia offers the benefit of enhanced muscle relaxation, potentially aiding in catheter removal [2,9]. The algorithm outlining the management of an entrapped epidural catheter is depicted in Figure 4.

## Conclusions

Although epidural anesthesia is an effective and well-tolerated technique for pain relief following surgery, it is not without potential complications. It is important to prevent these complications when possible and to appropriately manage them when they are encountered. Regarding entrapped epidural catheters, it is recommended to refrain from advancing epidural catheters more than 5 cm into the epidural space to minimize this potential complication. During the removal of all epidural catheters, patients should be carefully assessed for any neurological symptoms, and if such symptoms arise, manipulation of the catheter should cease immediately. It is advisable to maintain the same position used during catheter insertion while removing it to enhance procedural consistency and reduce the risk of complications. If a suspected entrapped epidural catheter is encountered, it is advisable to obtain imaging to aid in accurate diagnosis and enable appropriate management.
